# Expanded CCUG repeat RNA expression in *Drosophila* heart and muscle trigger Myotonic Dystrophy type 1-like phenotypes and activate autophagocytosis genes

**DOI:** 10.1038/s41598-017-02829-3

**Published:** 2017-06-06

**Authors:** Estefania Cerro-Herreros, Mouli Chakraborty, Manuel Pérez-Alonso, Rubén Artero, Beatriz Llamusí

**Affiliations:** 1 Translational Genomics Group, Incliva Health Research Institute, Valencia, Spain; 2 Department of Genetics and Interdisciplinary Research Structure for Biotechnology and Biomedicine (ERI BIOTECMED), University of Valencia, Valencia, Spain; 3 CIPF-INCLIVA joint unit, Valencia, Spain

## Abstract

Myotonic dystrophies (DM1–2) are neuromuscular genetic disorders caused by the pathological expansion of untranslated microsatellites. DM1 and DM2, are caused by expanded CTG repeats in the 3′UTR of the *DMPK* gene and CCTG repeats in the first intron of the *CNBP* gene, respectively. Mutant RNAs containing expanded repeats are retained in the cell nucleus, where they sequester nuclear factors and cause alterations in RNA metabolism. However, for unknown reasons, DM1 is more severe than DM2. To study the differences and similarities in the pathogenesis of DM1 and DM2, we generated model flies by expressing pure expanded CUG ([250]×) or CCUG ([1100]×) repeats, respectively, and compared them with control flies expressing either 20 repeat units or GFP. We observed surprisingly severe muscle reduction and cardiac dysfunction in CCUG-expressing model flies. The muscle and cardiac tissue of both DM1 and DM2 model flies showed DM1-like phenotypes including overexpression of autophagy-related genes, RNA mis-splicing and repeat RNA aggregation in ribonuclear foci along with the Muscleblind protein. These data reveal, for the first time, that expanded non-coding CCUG repeat-RNA has similar *in vivo* toxicity potential as expanded CUG RNA in muscle and heart tissues and suggests that specific, as yet unknown factors, quench CCUG-repeat toxicity in DM2 patients.

## Introduction

Myotonic dystrophy type 1 (DM1) and type 2 (DM2) are dominantly-inherited multi-systemic genetic disorders. DM1 (OMIM: 160900) is caused by an unstable expansion of a CTG trinucleotide repeat motif located in the 3′ untranslated region (UTR) of the *dystrophia myotonica protein kinase* (*DMPK*) gene^1^. Unaffected individuals carry fewer than 37 triplet-repeats, whereas expansions ranging between 50 and 4000 CTG repeats have been found in affected individuals. DM2 (OMIM: 602668), initially named proximal myotonic myopathy due to the greater weakness of proximal compared to distal muscles^2^, is caused by a tetranucleotide (CCTG) expansion in intron 1 of the CCHC-type zinc finger nucleic acid binding protein gene (*CNBP*, *also known as ZNF9*)^3^. Healthy individuals carry fewer than 30 tetra-nucleotide repeats, whereas repeat lengths found in affected patients are significantly longer than in DM1 (between 55 and 11000)^3^. In contrast to DM2, which does not have a congenital form, very large (>1,000 repeat) *DMPK* CTG mutations also cause congenital DM1 (CDM) characterized by neonatal hypotonia (floppy baby) and intellectual disability^4^. The expansions are transcribed into (CUG)n and (CCUG)n-containing RNA, respectively, which form secondary structures and sequester RNA-binding proteins, such as the RNA processing factors Muscleblind-like proteins (MBNL1-3 in vertebrates, Muscleblind in *Drosophila*), forming nuclear aggregates known as foci^5–11^. Additional splicing factors, such as CUGBP Elav-like family member 1 (CELF1), are also disrupted, leading to the mis-splicing of a large number of downstream genes^12–14^. Among them, the alteration in the splicing pattern of *CLCN1*, *INR*, *PKM*, *CACNA1S*, and *BIN1* pre-mRNAs has been associated with myotonia, insulin resistance, perturbed glucose metabolism and muscle weakness, respectively, which are all symptoms of DM^15–19^. Importantly, the repeat-length extensively correlated with disease severity in DM1^20^ and with the amount of MBNL sequestered in both types of DM^5, 21^. Although for DM2 the correlation between repeat length and disease severity in humans is less clear-cut, expression of non-coding CCUG-expanded RNA in flies has been shown to cause length-dependent toxicity in *Drosophila* eyes^22^.

Clinically, DM2 patients generally experience a milder phenotype than DM1 patients, including slower and less severe progression of the disease, reduced severity of the cardiac involvement with a significant reduction in arrhythmicity and prophylactic pacing requirements, lack of prominent late respiratory or facial and bulbar muscle weakness, less evocable myotonia, and preserved social and cognitive abilities^23–26^. However, the molecular origin of these milder phenotypes in DM2 is unknown. Indeed, several studies have reported that DM2 individuals tend to carry significantly more (75 to approximately 11,000, with a mean of 5,000 CCTG) repeats in mutant alleles compared to patients with CTG expansions (classic DM1 range is 100–1000 repeats)^3^ and, according to different sources *CNBP* is 4 to 8-fold more expressed in human muscles than the *DMPK* gene^27–29^. In addition, MBNL binds to CCUG with higher affinity than to CUG repeats^6, 30^, resulting in larger ribonuclear inclusions in DM2 patients, which sequester more MBNL^21^. Considering that CNBP is expressed at higher levels than DMPK in muscles, and that expanded alleles tend to carry more CTG repeats, as well as the fact that MBNL proteins have higher affinity for CCUG repeats than for CUG RNA, DM2 symptoms should be more severe, rather than milder, than DM1.

To investigate this paradox, we reasoned that the phenotypes brought about by both expansion types in *Drosophila* tissues might be informative. Significantly, weaker phenotypes are expected for CCUG expansions should they be intrinsically less toxic than CUG repeats, whereas similar phenotypes are expected if toxicity is modulated in humans by CCUG-specific factors. With this aim, we generated and characterized *Drosophila* models of DM1 and DM2 expressing pure CUG or CCUG repeats, respectively, in muscular and cardiac tissues. We found common pathogenic events between CUG and CCUG repeat toxicity, such as Mbl sequestration in foci, mis-splicing and increased autophagy in both tissues. Importantly, the severity of the phenotypes in the DM2 flies reveals that CCUG repeat expansions are potentially as toxic as CUG repeats in muscle and heart. Our study therefore suggests that unknown molecular RNA-toxicity modifiers account for the milder symptoms of DM2.

## Results

### Expression of either CUG or CCUG-expanded repeats sequester Muscleblind in ribonuclear foci in muscle and cardiac tissue

To accurately model DM1 and DM2 in flies, we generated *UAS-CTG* and *UAS-CCTG* transgenic fly lines carrying either 250 CTG (CTG (250)×) or 1100 CCTG (CCTG (1100)×) pure repeats, which are within the pathological range of repeat lengths and mimic the, at least 4 times longer, expansion size in DM2 patients compared with DM1^25, 31^. As controls, we generated flies carrying short versions of the repeats (CTG (20)× or CCTG (20)×). In order to express the repeats in different tissues, we crossed the UAS fly lines with the muscle-specific driver myosin heavy chain *Mhc-Gal4* ^32^ or the cardiac-specific driver *GMH5–Gal4* ^33^. The expression level of the repeats was assessed by qPCR using primers against the common SV40 terminator contained in these vectors (Fig. S1).

**Figure 1:**
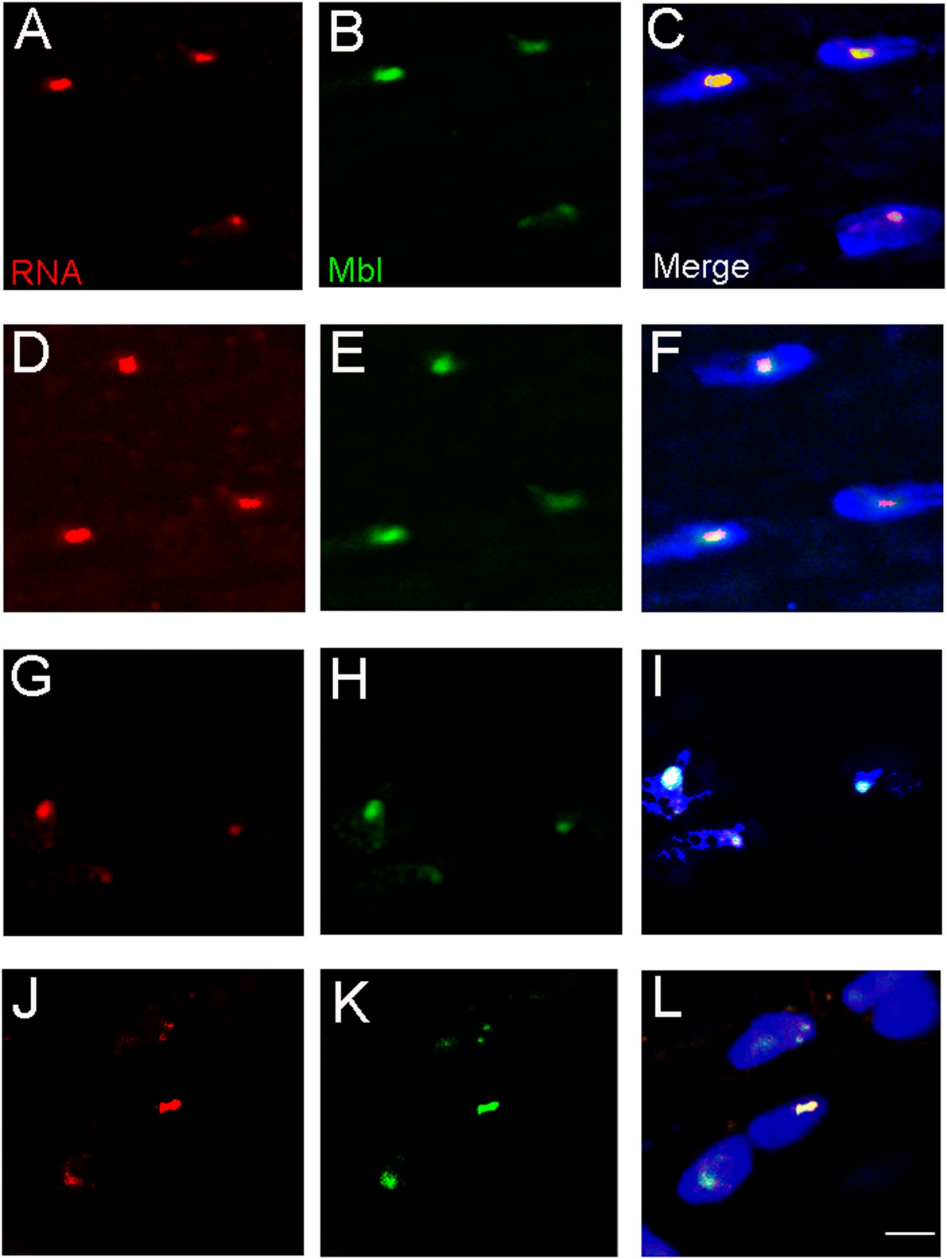
Muscleblind is retained in ribonuclear foci in flies expressing expanded CUG or CCUG repeats. Representative fluorescent confocal images of IFMs (**A**–**F**) and heart cells (**G**–**L**) from flies expressing expanded CUG (**A**–**C** and **G**–**I**) or CCUG (**D**–**F** and **J**–**L**) repeats under the control of the Mhc-Gal4 and GMH5-Gal drivers, respectively. Ribonuclear foci retaining Muscleblind were present in flies expressing long CUG or CCUG repeats. Merged images in (**C**,**F**,**I** and **L**) include DAPI (blue) counterstaining of the nuclei. Scale bar = 10 µm.

Fluorescent *in-situ* hybridization (FISH) to detect ribonuclear foci showed that they were present in the nuclei of indirect flight muscle (IFM) and heart cells expressing long CUG or CCUG repeats, but not in flies expressing the short versions of the repeats (Figs 1 and S2). Because Muscleblind sequestration is one of the main features of the disease, we studied Muscleblind subcellular localization in our model flies. As we previously reported, *Drosophila* Muscleblind is found in sarcomeric bands in adult muscle tissue and dispersed throughout the nuclei of cardiomyocytes^34, 35^. Muscleblind immunodetection in muscle and heart tissue in flies expressing the short versions of the CUG or CCUG repeats showed that Muscleblind localization was the same as that described in control samples. In contrast, Muscleblind was concentrated in CUG or CCUG ribonuclear foci in muscle and heart cells from flies expressing long CUG or CCUG repeats (Fig. 1). Thus, both expanded CUG and CCUG arrays originate ribonuclear foci and Muscleblind sequestration in *Drosophila* muscle and heart tissue, which are both histological hallmarks of DM.

### Muscleblind-dependent splicing is altered in flies expressing expanded CUG or CCUG repeats

**Figure 2:**
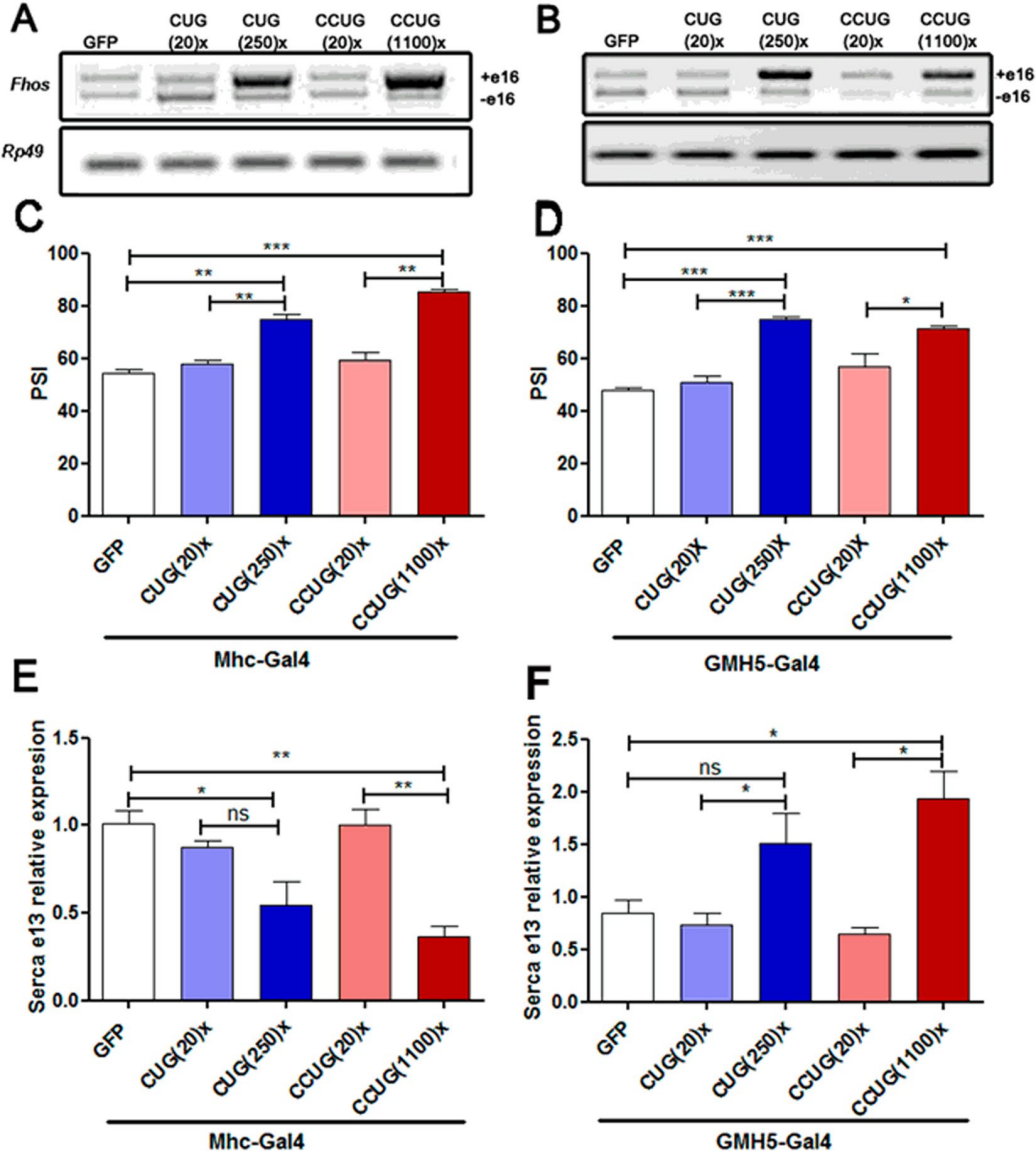
CUG and CCUG expansions cause Muscleblind-dependent missplicing. (**A**,**B**) Representative semi-quantitative RT-PCR showing inclusion of *Fhos* exon 16′ in flies expressing the indicated constructs in muscle (**A**) or heart (**B**) under the control of Mhc-Gal4 or GMH5-Gal4, respectively. Endogenous *Rp49* was used for normalization. Percentage of exon 16′ inclusion, revealed that expression of long CUG or CCUG repeats in the fly muscle (**C**) or heart (**D**), favored increased use of this exon. qRT-PCR results of *Serca* exon 13 expression relative to *Rp49 ﻿expression*, confirmed that the use of this exon in the flies expressing the expanded repeats and the control flies is significantly different in muscle (**E**) and heart tissues (**F**). The histograms show the mean ± SEM. *p < 0.05, **p < 0.01, ***p < 0.001 (Student’s t-test).

To test whether the confirmed Muscleblind retention in foci was enough to cause splicing misregulation, we studied the percentage of exon retention (“*percentage spliced in*”, PSI) of the *Drosophila formin* (*Fhos*) gene exon 16′, which has a highly conserved ortholog in human, with altered splicing in DM1 patients^36^. *Fhos* has 19 exons, which produce nine different transcripts (Ensembl Genome browser, release 83). We recently reported that exon 16′ (132 nt) is preferentially included in DM1 model flies expressing 480 interrupted CUG repeats (i(CUG)480) in muscle^37^. Importantly, this splicing event was shown to be Muscleblind-dependent. In control flies, the PSI of *Fhos* exon 16′ was around 50%. However, in flies expressing i(CUG)480 in muscle, this percentage increased to nearly 95%. Consistent with the milder toxic effects reported in DM1 individuals and in animal models with shorter CUG repeats, the inclusion percentage dropped to close 75% in flies expressing 250 CUG repeats in muscle. In the case of flies expressing expanded CCUG repeats in muscle, we also observed increased *Fhos* exon 16′ inclusion, which reached 85%. Importantly, flies expressing short versions of either CUG or CCUG repeats, showed no significant changes in exon usage (Fig. 2A and C). In cardiac tissue, the 50% exon inclusion found in control or short-repeat-expressing flies, increased to 75% in both expanded CUG and CCUG-expressing flies (Fig. 2B and D). We also quantified the inclusion of exon 13 of the Mbl-dependent *Serca* gene, which decreased 50% in the flies expressing the long repeats in muscle, while in heart, resulted into a 50% increase. Accordingly, in previous studies the expression of 480 interrupted CUG repeats in adult flies using the late muscle driver Mhc-Gal4 induced a 2.4-fold reduction of *Serca* transcripts with exon 13^37^, while the expression of 960 CUG repeats using the Mef-Gal4 driver resulted in increased expression^38^, suggesting a remarkable developmental-dependent regulation of this event in flies (Fig. 2E and F).

These data confirmed that the Muscleblind sequestration in ribonuclear foci observed in fly models of DM1 and DM2, led to a functional depletion of Muscleblind in adult muscle and heart tissue.

### The expression of autophagy-related genes is increased in muscular and cardiac tissues in DM1 and DM2 model flies

**Figure 3:**
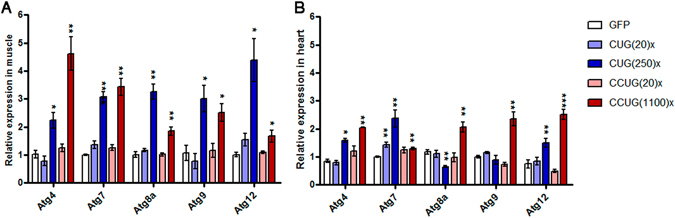
The expression of autophagy-related genes is upregulated in flies expressing expanded CUG or CCUG repeats in muscle or heart. Relative expression levels of *Atg4*, *Atg7*, *Atg8a*, *Atg9* and *Atg12* measured by qRT-PCR in muscle (Mhc-Gal4 driver; **A**) and heart samples (GMH5-Gal4 driver; **B**), showed a significant upregulation of these autophagy-related genes in flies expressing expanded CUG (CUG(250)×) or CCUG repeats (CCUG(1100)×). The histograms show the mean ± SEM. *p < 0.05, **p < 0.01, ***p < 0.001 (Student’s t-test).

Several studies have reported a pathological over-activation of the autophagy-lysosome pathway in DM1 models. Apoptotic activation and increased presence of autophagy markers has been reported in primary human cell lines from adult-onset DM1 patients^39, 40^ and in human DM1 embryonic stem cells-derived neural stem cells^41^. In addition, pathway analysis on global PolyA-seq studies of human DM skeletal muscle^42^ and brain^43^ identified enriched terms associated with ubiquitin-mediated proteolysis and the mTOR pathway. More recently, studies performed in a murine model of DM1 have reported that targeting deregulated AMPK/mTORC1 pathways improves muscle function in DM1^44^. Accordingly, we have previously demonstrated over-activation of apoptosis and autophagy by inducible expression of 480 interrupted CUG repeats in *Drosophila* adults and a rescue of muscle atrophy by silencing the expression of the autophagy-related genes Atg4, Atg7, Atg8a and Atg9^34^. To study the expression of autophagy-related genes in our DM1 and DM2 *Drosophila* models, we performed qPCRs with cDNAs from heart and thorax samples of flies expressing short and long versions of the CUG or CCUG repeats in heart and muscle (Fig. 3). In general, we found that expression of Atg4, Atg7, Atg8a, Atg9 and Atg12 were significantly upregulated in flies expressing either expanded CUG or CCUG repeats in muscle, compared to control flies expressing GFP or short repeats. Of note, the expression levels of these genes in flies expressing short CUG or CCUG repeats were similar to the levels in control flies that did not express the repeats (Fig. 3A). In comparison, the expression of the repeats in heart caused a moderate upregulation of *Atg* genes expression, in the case of flies expressing the long repeats. Upregulation of Atg genes mediated by the repeats was higher in the flies expressing CCUGs compared to those expressing CUG repeats (Fig. 3B). Consistent with these findings, we observed upregulation of *AKT2*, *AKT1S1* and *ATG4* mRNAs in human patient skeletal muscle^34^. These data support a role of autophagy activation in DM pathogenesis not only in DM1, as we previously reported, but also in DM2. In addition, our results highlight the relevance of the activation of this pathway in different tissues affected by repeat expression.

### Both expanded CUG and CCUG repeat RNA reduced cross-sectional muscle area and fly survival

**Figure 4:**
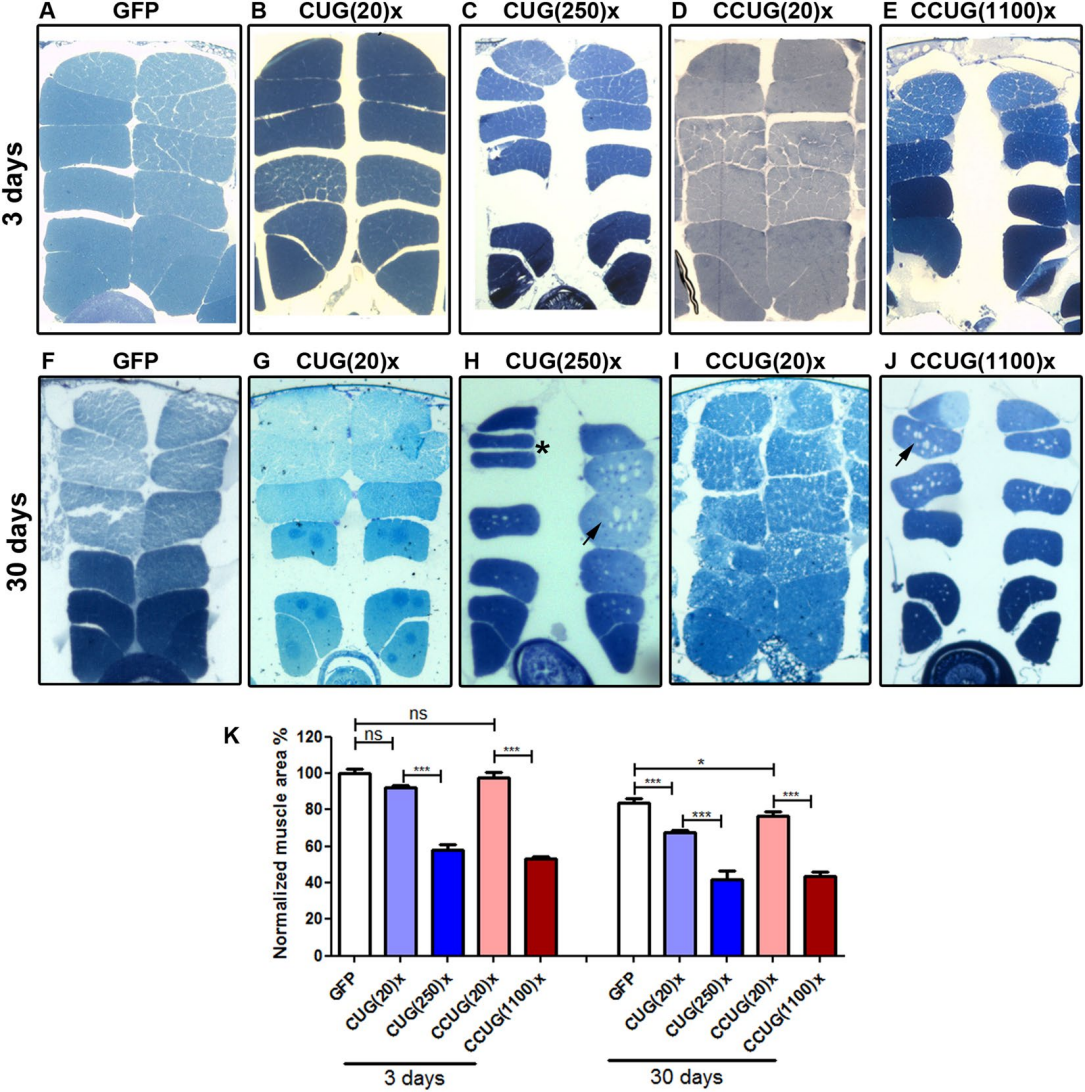
Expression of expanded CUG or CCUG repeats in muscle induces similar levels of muscle area reduction and degeneration. (**A**–**J**) Dorsoventral sections of resin-embedded fly thoraces. In all images the dorsal side is displayed at the top. Mhc-Gal4 was used to drive the expression of the indicated constructs in muscle. (**K**) Quantification of the mean percentage of muscle area per genotype relative to the muscle area of the control flies (GFP), which is considered as 100%. While young flies (3 day-old, in **A**–**E**) expressing 20 CUG or CCUG repeats were not different from control flies expressing GFP, flies expressing expanded CUG or CCUG repeats have a 50% reduction in IFM muscle area. All aged flies (30 day-old, in **F**–**J**) displayed reduced muscle area compared to young flies of the same genotype. However, vacuolization (arrows) and occasional muscle splitting (asterisk) characteristic of degenerating muscles were present only in muscles expressing expanded repeats. The graph shows the means ± *SEM*. *p < 0.05, **p < 0.01, ***p < 0.001 (Student’s t-test).

Despite the fact that MBNL1 is sequestered in CCUG foci and it is expected that the longer CCUG repeat expansions will have a greater inhibitory effect on MBNL1 in DM2 cells, visible muscle atrophy in DM2 muscle is actually milder than in DM1 patients^45^. To investigate how *Drosophila* muscle responds to expanded CCUG repeat RNA, we quantified the cross-sectional muscle area of IFMs from adult flies at different ages that expressed 250 CUG or 1100 CCUG repeats, or controls expressing 20 units or the GFP reporter, under the control of the Mhc-Gal4 driver. We observed a significant reduction in muscle area in 3-day-old flies expressing long CUG or CCUG repeat RNA, whereas cross-sectional muscle area in flies expressing the short versions of the repeats were not significantly different from control GFP-expressing flies. Importantly, flies expressing either long CUG or CCUG repeats showed similar muscle phenotype, which reached up to a 50% reduction in muscle area in both cases (Fig. 4A–E and K). Similarly, muscle area in aged flies (30-day-old flies) expressing expanded repeats was reduced in comparison to aged GFP flies. The decrease in the muscle area in young and aged flies was similar in all the genotypes studied (around 20%) suggesting that the strong muscle reduction observed in the model flies had an important developmental component. Nevertheless, we observed vacuolization, splitting muscles and occasional absence of muscle packages, characteristic of degenerating muscles^46^, which were only present in aged flies expressing the expanded CUG or CCUG repeats (Fig. 4F–K). Taken together, these results suggests that toxic RNAs interfere with both muscle development and muscle maintenance.

**Figure 5:**
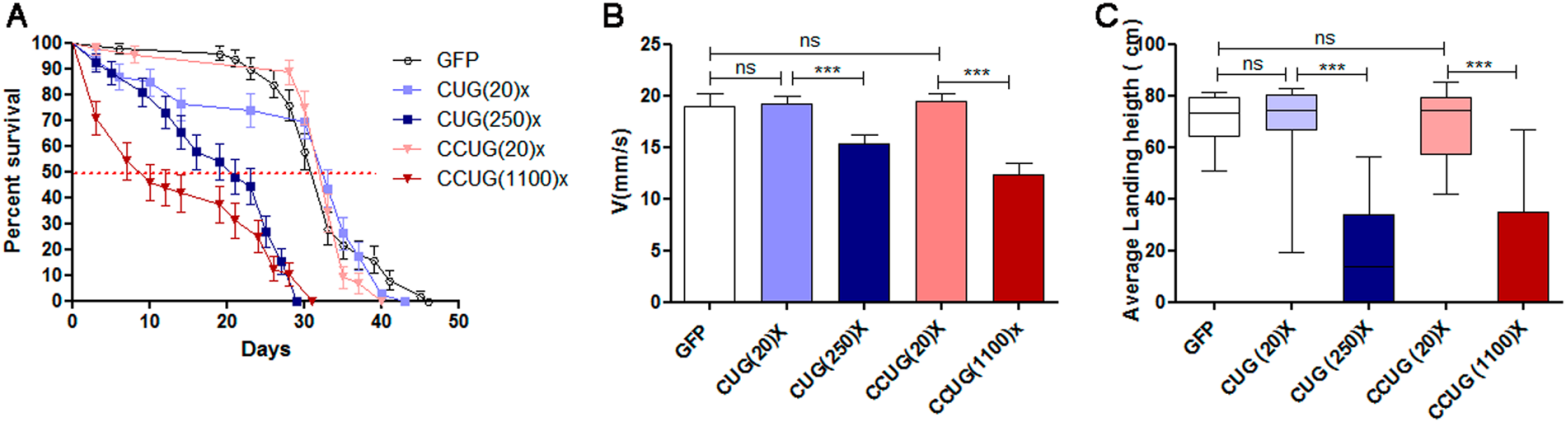
Survival and locomotor function were reduced in flies expressing expanded CUG or CCUG repeats in muscle. (**A**) Average percentage of live flies versus age (in days). The Mhc-Gal4 driver was used to induce the expression of the indicated constructs in muscle. The horizontal dotted line marks the median survival. Whereas control and short-repeat-expressing flies had similar median survival (GFP; n = 90, CUG(20)×, n =  100 and CCUG(20)×, n = 95), long CUG and CCUG-expressing flies have reduced survival (CUG(250)×; n =  95 and CCUG(1100)×; n = 100). Differences in the survival curves were highly significant (p < 0.0001, log-rank test). (**B**) Histogram showing the climbing speed as the mean speed ± *SEM* in mm/s. Flies expressing long CUG or CCUG repeats had reduced climbing velocity compared to control flies or flies expressing the short versions of repeats. (**C**) Notched box plot showing the median and the distribution of the average landing height data obtained in the flight assay with the relevant genotypes. Flight disability was observed in flies expressing long CUG or CCUG repeats. *p < 0.05, **p < 0.01, ***p < 0.001 (Student’s t-test).

Population studies have reported higher mortality and morbidity rates, and a positive correlation between the age at onset of DM1 and age at death in patients^47, 48^. Similarly, we observed that the lifespan and mean survival of flies expressing expanded CUG or CCUG repeat RNA was significantly reduced in comparison to control flies expressing only GFP, whereas the lifespan of flies expressing 20 units of the repeats was not significantly different from the control flies (Fig. 5A). These results are consistent with our previous description of muscle loss, degeneration and reduced viability of flies expressing i(CTG)480 throughout the fly musculature^34, 49^. Taken together our data indicate that the expression of expanded CUG or CCUG repeats in muscle causes similar defects in the IFMs of young and aged flies, and in the viability of *Drosophila*.

### Locomotor performance is compromised in flies expressing expanded CUG or CCUG repeat RNA in muscle

To test whether the muscle loss observed in the model flies was of functional relevance, we assessed the flight and climbing ability of flies expressing the expanded repeats and compared them to control flies expressing GFP or short repeats. Climbing velocity and landing distance were only reduced in flies expressing the expanded versions of the repeats and no significant differences were observed between DM1 and DM2 model flies. Of note, these functional parameters were not altered in flies expressing the short versions of the repeats ﻿compared to the controls. In the case of climbing velocity, flies expressing the long CUG or CCUG repeats retained 70% of the control-fly climbing speed, and there was no significant difference in velocity between these two genotypes (Fig. 5B). The average landing height was reduced to 25% compared to control flies expressing GFP or 20 units of the repeats, and was similar in flies expressing either expanded CUG or CCUG repeat RNA (Fig. 5C). Thus, in contrast to human patients, where DM2 muscle disability is milder than in DM1, these data indicate that expression of long CUG or CCUG repeat RNA in muscle tissue has a similar effect on locomotion in flies.

### Heart dysfunction in both DM1 and DM2 model flies includes systolic and diastolic alterations, arrhythmia, and contractility defects

**Figure 6:**
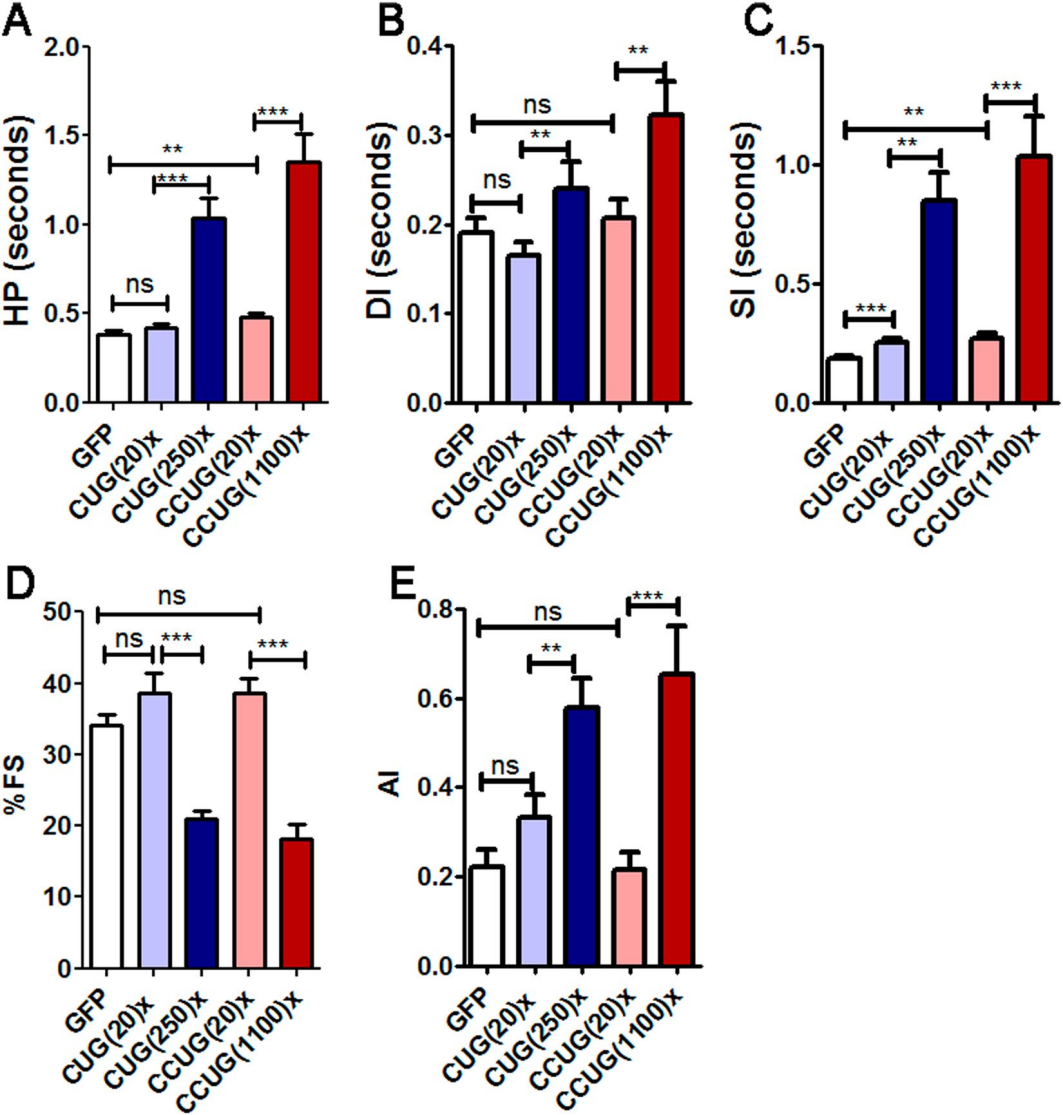
Cardiac dysfunction in DM1 and DM2 model flies includes diastolic and systolic elongation, increased arrhythmicity, and reduced contractility. The mean heart period (HP, in **A**) was significantly increased in flies expressing expanded CUG or CCUG repeats in heart. This increase was caused by a prolongation of both diastolic and systolic intervals (DI, in **B** and SI, in **C**) in the model flies. Heart tube contractility and cardiac rhythm were also affected in these flies, because the percentage of fractional shortening (%FS in **D**) was reduced to only 20% and arrhythmia, measured as the arrhythmicity index (AI in **E**), was significantly increased to similar levels in both DM1 and DM2 model flies. Graph bars show the mean values and their standard errors (n = 18 to 29). *p < 0.05, **p < 0.01, ***p < 0.001 (Student’s t-test).

Cardiac alterations, characterized by conduction delays, arrhythmia, and heart blockage are the second most common cause of death in DMs^50^. In DM2, cardiac abnormalities have been reported to be similar to those described in DM1 but less frequent and severe^24^. To study heart function in the *Drosophila* DM models, adult fly hearts were dissected in artificial hemolymph and recorded with a high-speed video camera. Cardiac contractions were analyzed using a semi-automatic optical heartbeat analysis (SOHA) method to quantify the fly heart functional parameters^51^. The study of heart function in DM2 model flies revealed that expression of long CCUG repeats in fly heart caused lengthening of the heart period (HP), and extension of the systolic and diastolic intervals (SI and DI, respectively). Heart contraction, measured as a percentage of fractional shortening (%FS), and arrhythmicity measured using the arrhythmia index (AI), were significantly altered compared to controls. In these model flies, the %FS was reduced to 20% and AI increased by around 3-fold (Fig. 6D,E). Importantly, the expression of short CCUG repeats did not affect %FS or AI but it increased the SI compared to controls, and resulted in a significantly increased HP (Fig. 6A–C). Similarly, we previously reported, that overexpression of expanded CUG repeats *in Drosophila* heart results in an increased HP with prolonged DI and SI, a reduction in %FS, and increased AI. In contrast, the expression of short CUG repeats only produced a slight increase in the SI duration^35^.

### Expression of expanded CUG or CCUG repeat RNA in fly heart reduces survival but does not affect locomotion

**Figure 7:**
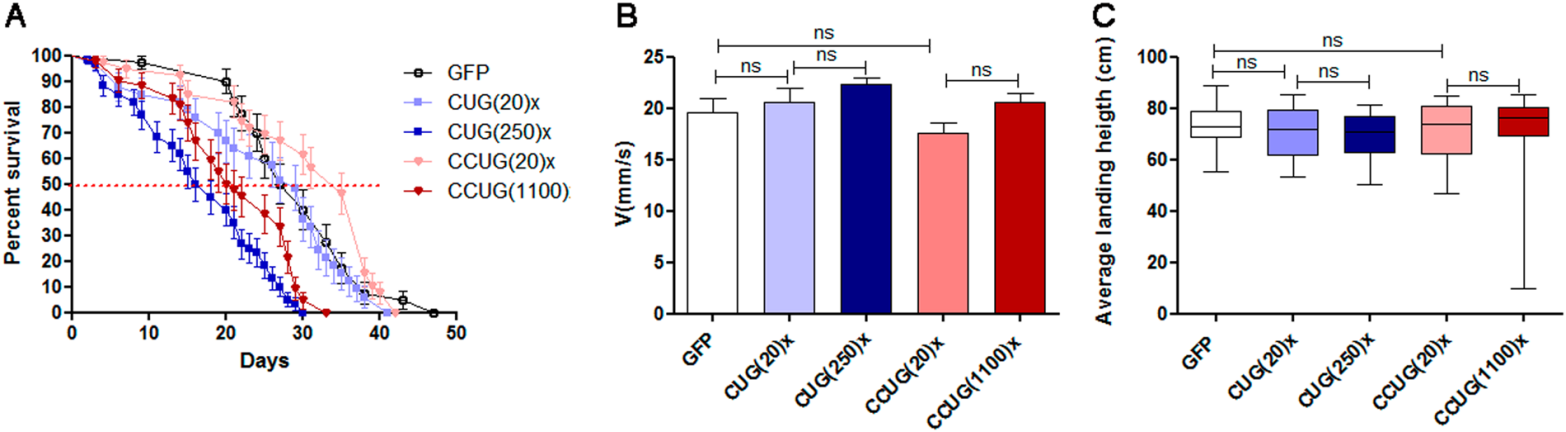
Expression of expanded CUG or CCUG repeats in fly heart alters survival but not locomotion. (**A**) Average percentage of live flies, with the indicated genotypes, versus age (in days). The *GMH5-Gal4* driver was used to induce expression of the indicated genotypes in cardiomyocytes. Horizontal dotted line marks the median survival. Flies expressing expanded CUG (CUG(250)×; n = 100) or CCUG (CCUG(1100)×; n = 97) repeats had a reduced lifespan compared to control flies (GFP; n = 100) or flies expressing the short versions of the repeats (CUG(20)×; n = 95 and CCUG(20)×; n = 99). The differences in survival curves were highly significant (p < 0.0001, log-rank test). (**B**) Histogram showing the climbing velocity of flies as the mean speed ± *SEM* in mm/s. Expression of long CUG or CCUG repeats in heart did not modify climbing velocity compared to control flies or flies expressing the short versions of repeats. (**C**) Notched box plot showing the median and the distribution of the average landing height data obtained in the flight assay using flies with the genotypes indicated. *p < 0.05, **p < 0.01, ***p < 0.001 (Student’s t -test).

We previously reported that overexpression of long CUG repeats in fly heart results in a reduction in mean survival and lifespan^35^. The mean survival in control flies expressing GFP was 29 days which was reduced to about half in the DM1 model flies. The survival curve for flies expressing CCUG repeats in heart tissue was also significantly reduced compared to the GFP control flies. Of note, the survival curve of flies expressing short CUG or CCUG repeats was similar to that of control flies (Fig. 7A). These data suggest that the cardiac alterations in our DM1 and DM2 models affect the survival of flies.

To assess whether the expression of repeats in heart affects locomotor performance in flies, we analyzed the climbing velocity and landing distance of flies expressing CUG or CCUG repeats and found that neither the expression of short nor long versions of CUG or CCUG repeats affected these abilities (Fig. 7B,C). Thus, the reduction in %FS did not affect acute workload demands (flight, and climbing), but did have an accumulative detrimental effect on survival.

## Materials and Methods

### Drosophila strains

Pure expanded CTG and CCTG repeats were generated by PCR amplification of self-priming single-stranded CTG and CAG or CCTG and CAGG oligonucleotides as previously described^52^. Synthesized DNA duplexes were electrophoresed, size fractionated, purified using a DNA gel extraction kit (Qiagen), 5′-phosphorylated with T4 polynucleotide kinase, and cloned into the *Eco*RV site of pUAST. The recombinant plasmids containing uninterrupted stretches of CTG or CCTG repeats were amplified in STBL3 *E*. *coli* (Invitrogen) at 20 °C. Plasmid DNA was purified using a Qiagen plasmid DNA purification kit and sequenced from both ends to ensure the sequence integrity of the clones. Transgenic flies were generated by injecting the plasmids into *w* ^*1118*^ embryos by BestGene Inc. following the method described in ref. 53. UAS-GFP strain was obtained from the Bloomington *Drosophila* Stock Center (Indiana University, Bloomington, IN). The cardiomyocyte-specific driver *GMH5–Gal4* was kindly provided by Dr. Bodmer from the Sanford Burnham Institute, California, USA^33^. The Mhc-GAL4 line was previously described^32^. Mhc-Gal4 drives expression in terminally differentiated muscle under the control of endogenous myosin heavy chain regulatory regions, while GMH5-Gal4 is expressed in cardiomyocytes initially driven by a 900 nt *tinman* heart enhancer and later maintained by a *UAS-Gal4* autoregulatory loop^33^. All the fly lines were maintained in standard *Drosophila* food. The flies were grown at 25 °C to study the effect of expressing repeats throughout the musculature and at 29 °C to study the cardiac defects. Expression levels of the different transgenes were assessed as previously described^35^.

### Cardiac physiological analysis

For the physiological analysis, female flies were collected just after eclosion and were maintained for 7 days at 29 °C. For the heart-beat recordings, semi-intact heart preparations were made as previously described^54, 55^. An Leica DFC 450C microscope, connected to an ORCA Flash (Hamamatsu) high-speed digital camera was used to take 20 s recordings at a minimum speed of 150 frames/s. Different cardiac parameters were measured using SOHA software^51^.

### Histological analysis

Analysis of the IFM area in *Drosophila* thoraces was performed as previously described^56^. Briefly, six thoraces from three-day or thirty-day-old (aged group) females were embedded in Epon following standard procedures. After drying the resin, semi-thin 1.5 µm-sections were obtained using an ultramicrotome (Ultracut E, Reichert-Jung and Leica). Images were taken at 100× magnification with a Leica DM2500 microscope (Leica Microsystems, Wetzlar, Germany). To quantify the muscle area, five images containing IFMs per fly were converted into binary images. Considering the complete image as 100% of the area, we used ImageJ software to calculate the percentage occupied by pixels corresponding to the IFMs. The percentage of pixels occupied by muscle in the control GFP flies were considered as 100%, and the percentage of muscle area of the rest of genotypes were normalized to these control flies.

For immunofluorescence analysis, dissected fly hearts or *Drosophila* thorax longitudinal sections were fixed for 20 min in 4% paraformaldehyde, and washed in PBT (PBS containing 0.3% Triton X-100) before staining. Muscleblind staining, and FISH to detect ribonuclear CUG foci, were performed as previously described^56^. The specificity of the anti-Mbl antibody has been previously tested in ref. 56. To detect CCUG foci a variation of the FISH protocol was implemented using a locked nucleic acid (LNA) probe with 7 CAGG repeats (Sigma), which was hybridized at 55 °C. All the confocal images were taken with an Olympus FV1000 microscope.

### RNA extraction, RT-PCR and qRT-PCR

For each biological replicate, total RNA was extracted using Trizol (Sigma) from 10 five-day old adult males for the muscle studies and 20 seven-day old adult female hearts for the cardiac studies. One microgram of RNA was digested with DNase I (Invitrogen) and reverse-transcribed with SuperScript II (Invitrogen) using random hexanucleotides. 20 ng of cDNA were used in a standard PCR reaction with GoTaq polymerase (Promega) and specific primers to analyze *Fhos* exon 16′ inclusion (Table S1). *Rp49* was used as endogenous control using 0.2 ng of cDNA. qRT-PCR to analyze *Serca* exon 13 and *Atg 4*, *Atg7*, *Atg8a*, *Atg9* and *Atg12* expression levels was carried out from 2 ng of cDNA template with SYBR Green PCR Master Mix (Applied Biosystems) and specific primers (Table S1). For reference gene, *Rp49*, qRT-PCR was carried out from 0.2 ng of cDNA. Thermal cycling was performed in Step One Plus Real Time PCR System (Applied Biosystems). Three biological replicates and three technical replicates per biological sample were carried out. Relative expression to endogenous gene and the control group was obtained by the 2^−∆∆Ct^ method. Pairs of samples were compared using two-tailed t-test (α = 0.05), applying Welch’s correction when necessary.

### Survival curves

Survival experiments were performed independently twice with a minimum of 45 flies each time. Flies were maintained at 25 °C for experiments involving Mhc-Gal4 and at 29 °C for the GMH5-Gal4 driver. The flies were transferred to new fresh nutritive media every second day and scored for deaths daily.

### Flight and climbing functional assays

Given the heterogeneity generally found in the functional assays performed with female flies, we only used males in these experiments. Flight assays were performed on day five as described previously^57^ using 100 flies per group. To assess climbing velocity, groups of 15, five-day-old males were transferred into 25 cm long, 1.5 cm diameter pipettes, after a period of 24 h without anesthesia. The height reached from the bottom of the vial by each fly in a period of 10 s was recorded with a camera. For each genotype, approximately 30 flies were tested.

### Statistical analysis

Statistical analysis was performed using GraphPad Prism5 software. Pairs of samples were compared using a two-tailed Student’s t-test (α = 0.05), applying Welch correction when necessary. The survival curves used a minimum of 90 individuals and a log-rank test was used to assess whether there were any significant differences between them. The flight assay data are represented as a notched box plot, which includes the median and the distribution of the average landing heights obtained; the horizontal lines inside the boxes represent the median values, the bottom and top edges of the boxes represent the 25th and 75th percentiles, and bottom and top whiskers reach the 10th and 90th percentiles, respectively.

## Discussion

A significant feature of DM is that two different microsatellite expansions in two unrelated genes cause a clinically similar disease. The histological features of skeletal muscle biopsies taken from DM1 and DM2 patients are very similar^50, 58^. In both diseases, affected muscles show central nuclei, a reduction in the number and diameter of specific fiber types, fibrosis and adipose deposition. DM2 is specifically characterized by the presence of atrophic fibers with nuclear clumps even before the muscle weakness appearance as well as by a predominant type 2 fiber atrophy^59, 60^. In DM2, cardiac abnormalities have also been reported to be similar to those described in DM1, including conduction disturbances, cardiac arrhythmias and sudden death^26, 61–63^. Similarly, the characteristic features of DM that we describe in our DM2 (CCUG-repeat bearing) flies, including muscle and locomotor defects, cardiac dysfunction and reduced survival, were very similar to the characteristics of flies expressing CUG repeats. Interestingly, the phenotypic similarities between our DM1 and DM2 model flies go beyond phenotypes to the pathogenesis mechanisms. We showed that Mbl retention in foci resulting in missplicing, and autophagy activation are common to both diseases. We report that autophagy-related genes are upregulated not only in muscle, but also in heart in both models, suggesting that this is not a tissue-specific mechanism of repeat toxicity. Some important differences between both models are also highlighted in our study. The effect of the expression of long CCUG repeats in heart was more pronounced than that of long CUG repeats, and correlated with stronger upregulation of autophagy-related factors. These data suggest the existence of unknown tissue-dependent factors that might modulate the toxicity of CUG and CCUG repeats. The difference between the expression level of autophagy-related factors in control or expanded repeat-expressing flies was higher in muscle compared to heart samples, suggesting that autophagy is importantly involved in pathogenesis in this tissue. The autophagy activation in the DM1 and DM2 model flies coincides in muscle with strong muscle area reduction in the flies expressing the long versions of the repeats. These data are consistent with our previous results in the model flies expressing 480 CUG repeats^34^. Moreover, our experiments with young and aged flies have shown that muscle defects caused by expanded CCUG repeats have not only a developmental contribution but may also impinge on adult muscle maintenance and/or degeneration, as we have previously shown with heat-shock-induced expression of CUG expansions exclusively in adult muscle^34^. Importantly, this is the first DM2 animal model showing obvious muscle phenotypes.

As a result of expanded repeats expression in heart, we observed systolic and diastolic dysfunction, reduction of the fractional shortening and increased arrhythmicity in DM2 model flies, which resembled the DM1-like phenotype previously described in flies^35^ and in DM patients^64^. Importantly, SI and DI were more affected by CCUG repeats than by CUG repeats expression. Accordingly, in heart tissue, the expression of short repeats produced a slight but significant prolongation in the systolic interval, which was more pronounced in the case of CCUG-expressing flies. Remarkably, the expression of short versions of repeats did not induce Muscleblind sequestration in foci in IFM or heart tissue. Therefore, the phenotypes observed in these flies might be independent of Muscleblind, and the factors originating the phenotype seem to be more sensitive to CCUG repeats than to CUG repeats.

An open question in the field of DM is to clarify the pathomechanisms underlying the phenotypic differences between DM1 and DM2. Several studies have confirmed that the frequency and severity of cardiac involvement and of muscle weakness are reduced in DM2 compared to DM1 and that progression is slower and less severe in DM2^24, 26^. This suggests that other cellular and molecular pathways are involved besides the shared toxic-RNA gain of function in the human disease phenotype. Three factors have been shown to influence the level of toxicity of expanded repeats in the RNA; expression level, length, and sequence^21, 65, 66^. Longer sequences tend to cause severe pathogenesis but depending on the sequence, RNA binding factors might be differentially affected. Importantly, in DM2 patients, the severity of the disease has not been directly correlated with the repeat number, only a relationship between repeat lengths and MBNL1 rate of sequestration has been established^21^. In flies, however, a previous report showing the effect in eye of the expression of pure, uninterrupted CCUG-repeat expansions ranging from 16 to 720 repeats in length, has shown a nice correlation between length and toxicity of the CCUG repeats^22^. We believe that this previous observation, and our own reports of similar phenotypes in flies expressing either expanded CUG or CCUG repeats, suggest the existence of unknown modifiers in humans, which might quench RNA toxicity in DM2 patients.

In our flies expressing 250 CUG repeats we observed very similar phenotypes but milder than the ones previously reported by expressing 480 interrupted CUG repeats in muscle^34, 49^. Our data suggests that these phenotypes are sensitive to CUG repeat length, a main feature of DM1, and suggest that the phenotypes described in the previous model were not significantly affected by interrupting sequences.

The experiments expressing the CUG or CCUG repeats in a non-human context in *Drosophila* provide evidence of the strong toxicity potential of the CCUG repeats, as the phenotypes we report in the DM2 model flies expressing the repeats in muscle or heart, are as strong as the phenotypes obtained from expressing the CUG repeats. Disease-specific manifestations may then result from factors that are extrinsic to the repeats and previous evidence suggested several hypotheses. Disease-specific manifestations may result from differences in spatial and temporal expression patterns of *DMPK* and *CNBP* genes. Similarly, changes in the expression of neighboring genes may define disease-specific manifestations. It was recently reported that CUGBP1 protein is overexpressed in muscle biopsies from patients affected by the adult classical form of DM1 but not in muscle from DM2 patients, suggesting that CUGBP1 overexpression in DM1 might be an additional pathogenic mechanism not shared by DM2^67^. Another possible explanation for the clinical differences between the two DM forms is the reduction of DMPK or ZNF9 protein levels in DM1 and DM2 respectively^68–70^. However, *Dmpk* knockout young mice do not develop a multisystemic phenotype mimicking myotonic dystrophy^71^. On the contrary, reduction of *CNBP* levels is sufficient to produce multiorgan symptoms resembling those of DM as observed in heterozygous Cnbp +/− knockout mice^72^ implying that CNBP may well play a role in DM2 pathology^21^. According to different sources, *CNBP* is 4 to 8-fold more expressed in human muscles than the *DMPK* gene^27–29^, which makes it difficult to explain the phenotypic differences between DM1 and DM2 based on the small reductions in *CNBP* expression reported in DM2 patients. Another important difference between CUG and CCUG expansions is that MBNL has been reported to bind CCUG repeats with a stronger affinity compared to CUG repeats^6, 30^. In addition, the ribonuclear inclusions in DM2 patients appear to be larger than in DM1 patients, and sequester more MBNL^21^. Accordingly, our results in the DM model flies show that, at least in muscle, flies expressing expanded CCUG repeats tend to have higher levels of missplicing, suggesting a reduced activity of Mbl. However, the muscle defects in DM1 and DM2 model flies were similar, suggesting that Mbl involvement in muscle phenotype is already limiting in DM1 model flies and decreasing levels of Mbl would not result in stronger phenotype.

In conclusion, through this demonstration of CUG and CCUG repeat-induced toxicity in different fly tissues we have gained a useful insight into the differences and similarities in the mechanism of DM pathogenesis in these tissues. The dual system we report (DM1 vs DM2 fly model) with well-characterized repeat expression, resulting phenotypes and molecular alterations, will also be useful to compare the effect of potential chemical or genetic modifiers of RNA toxicity on each of these diseases. The potential discovery of genetic modifiers that affect only one of the components in flies, either CUG or CCUG toxicity, could explain the clinical differences between both human diseases, contributing to increase the knowledge about their pathogenesis pathways and towards the development of new treatments.

## Electronic supplementary material

Supplementary information
