# Supplementary material for: Expanded CCUG repeat RNA expression in Drosophila heart and muscle trigger Myotonic Dystrophy type 1-like phenotypes and activate autophagocytosis genes
Source: Sci Rep. 2017 Jun 6;7:2843. doi: 10.1038/s41598-017-02829-3 (PMC5460254; doi:10.1038/s41598-017-02829-3)
Supplement: Supplementary file 1 — Supplementary information [file 41598_2017_2829_MOESM1_ESM.pdf]

**Expanded CCUG repeat RNA expression in Drosophila heart and muscle trigger Myotonic Dystrophy type 1-like phenotypes and activate autophagocytosis genes.**

Estefania Cerro-Herreros[1,2,3 $], Mouli Chakraborty[1,2,3$], Manuel Pérez-Alonso[1,2,3], Rubén Artero[1,2,3*], and Beatriz Llamusí[1,2,3]

## Supplementary Information

**Fig S1: Expression levels of the indicated constructs in muscle (A) and cardiac tissues (B) relative to the expression of the line expressing CUG(250)×.** qPCR to detect the expression of the common terminator SV40 showed that the expression of expanded CCUG repeats in muscle was 3-fold higher than the expanded CTG repeats. However, in heart, the expression levels of all four transgenes were similar. $*p < 0.05$, $**p< 0.01$, $***p < 0.001$ (Student´s t -test).

**Fig S2: Flies expressing short CUG or CCUG repeats have no ribonuclear foci containing Muscleblind.** Representative fluorescent confocal images of muscle (A-F) and heart cells (G-L) from flies expressing short CUG (A-C and G-I) or CCUG (D-F and J-L) repeats. Muscleblind was found in sarcomeric bands in muscles and dispersed in the nuclei of heart cells expressing short CUG or CCUG repeats and ribonuclear foci were not detected. Merged images in C, F, I and L include DAPI (blue) counterstaining of the nuclei. Scale bar = 10 µm.

Fig. S1

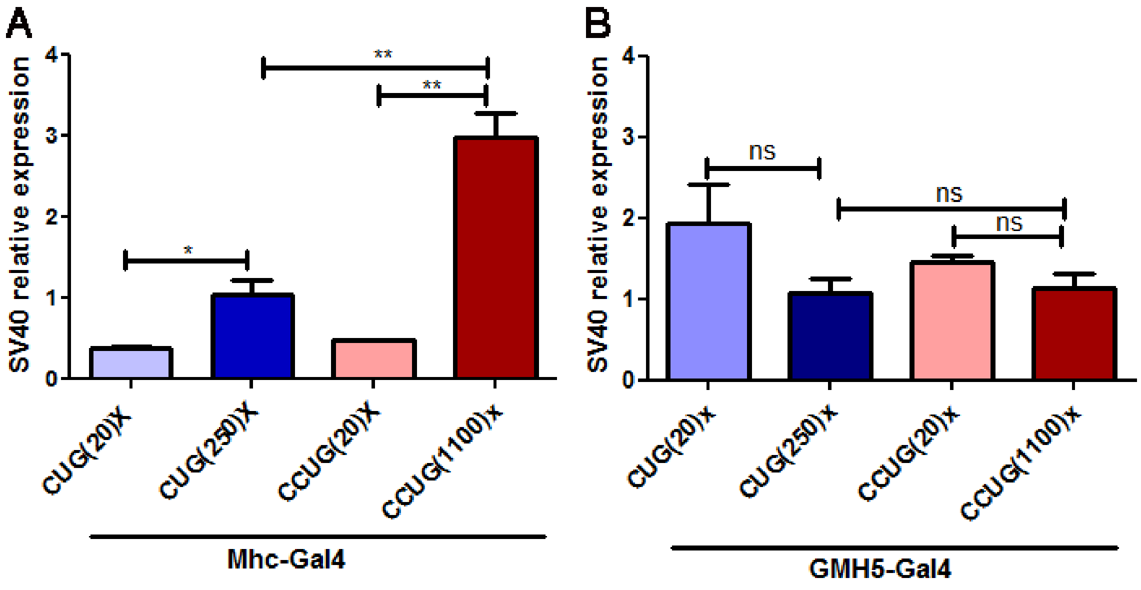
A
SV40 relative expression
0
1
2
3
4
*
**
**
CUG(20)X
CUG(250)X
CCUG(20)X
CCUG(1100)x
Mhc-Gal4
B
SV40 relative expression
0
1
2
3
4
ns
ns
ns
CUG(20)x
CUG(250)x
CCUG(20)x
CCUG(1100)x
GMH5-Gal4

**Fig. S2**

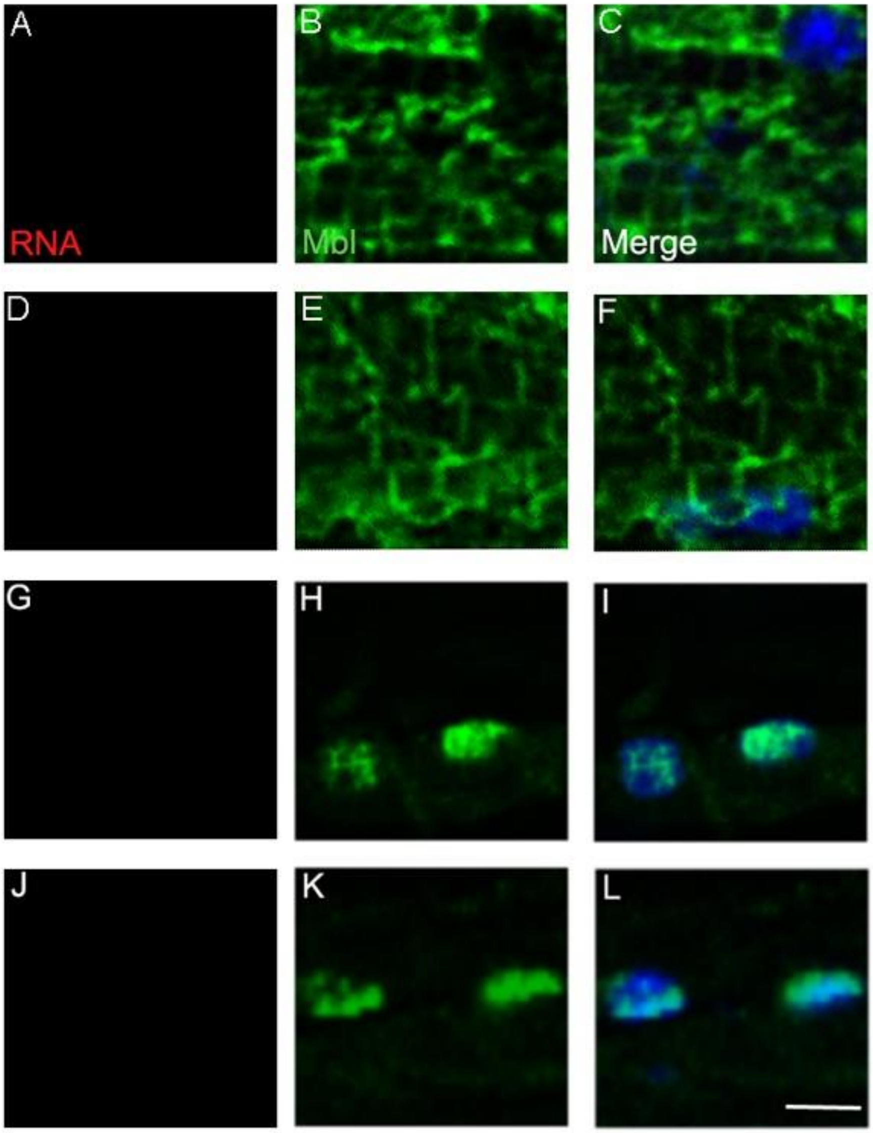

**Table S1. Oligonucleotide sequences**

| Primer | Sequence (5’ → 3’) | qRT-PCR/RT-PCR |
|---|---|---|
| *Atg4 fwd* | GCGCTCTTCGAGATCAGTCA | qRT-PCR |
| *Atg4 rev* | CCTGCCGCTCTCTTCAACTA | qRT-PCR |
| *Atg7 fwd* | CATAGCCTGTTCAGCGGCCGT | qRT-PCR |
| *Atg7 rev* | CCGCTTGAATTCGGAGATTCCCGTC | qRT-PCR |
| *Atg8a fwd* | ATCCAGACCGTGTGCCCGTCAT | qRT-PCR |
| *Atg8a rev* | ACCGACGGTCAGGTCGGAAGG | qRT-PCR |
| *Atg9 fwd* | CCACATCGAGGACCTTGACTC | qRT-PCR |
| *Atg9 rev* | CCACTACAATCACTGTGAATCCG | qRT-PCR |
| *Atg12 fwd* | TCGATGCCAGCGAGCAAATTTTCCT | qRT-PCR |
| *Atg12rev* | GCCCCACGCCTGATTCTTGCA | qRT-PCR |
| *Serca fwd* | GCAGATGTTCCTGATGTCG | qRT-PCR |
| *Serca rev* | CGTCCTCCTTCACATTCAC | qRT-PCR |
| *Rp49 fwd* | GGATCGATATGCTAAGCTGTCGCACA | qRT-PCR/ RT-PCR |
| *Rp49 rev* | GGTGCGCTTGTTCGATCCGTAACC | qRT-PCR/ RT-PCR |
| *Fhos fwd* | GTCATGGAGTCGAGCAGTGA | RT-PCR |
| *Fhos rev* | TGTGATGCGGGTATCTACGA | RT-PCR |
